# The Adler grade by Doppler ultrasound is associated with clinical pathology of cervical cancer: Implication for clinical management

**DOI:** 10.1371/journal.pone.0236725

**Published:** 2020-08-10

**Authors:** Dehong Che, Zhirong Yang, Hong Wei, Xuedong Wang, Jiayin Gao

**Affiliations:** 1 Department of Obstetrics and Gynecology, The Second Affiliated Hospital of Harbin Medical University, Harbin, China; 2 Department of Ultrasound, Harbin Red Cross Center Hospital, Harbin, China; 3 Department of Ultrasound, The Second Affiliated Hospital of Harbin Medical University, Harbin, China; 4 Department of Obstetrics and Gynecology, Longnan Hospital, Daqing General Hospital, Daqing, China; University of Vermont Larner College of Medicine, UNITED STATES

## Abstract

**Objective:**

To analyze the relationship of Adler grade by transvaginal color Doppler flow imaging (TV-CDFI) and the clinical pathological parameters of patients with cervical cancer, and to identify the value of Adler grade in the diagnosis and treatment of cervical cancer.

**Methods:**

Patients with cervical cancer diagnosed pathologically in our hospital from January 1, 2019 to December 31, 2019 were included, All patients underwent TV-CDFI examination, and the images were divided into 0 to III grades according to the Adler grades, and the correlations between the Adler classification and clinical pathological parameters (clinical stage, mass size, pathological type, squamous cell carcinoma subtype, CA125, CA199) were analyzed.

**Results:**

A total of 162 patients with cervical cancer were included. With the increase of Adler severity, the clinical stage of cervical cancer increased accordingly. the cancer size differed significantly in patients with different Adler grade (p = 0.004); There were significant differences in the level of CA125, CA199 between the squamous cell carcinoma and adenocarcinoma (all p<0.05). the Adler grade was positively related with the clinical stage, pathological type and squamous cell carcinoma subtypes of cervical cancer (all p<0.05), no correlations were found among the Adler grade and the cancer size, CA125, CA199 (all p>0.05). The area under ROC curve of the cervical squamous cell carcinoma predicted by Adler grade based on FIGO results and pathological results was 0.811and 0.762 respectively (all p<0.05).

**Conclusions:**

Adler grades are closely associated with the clinical pathology of cervical cancer, which may be a convenient and effective approach for the assisting assessment of cervical cancer.

## Introduction

Cervical cancer is one of the most common malignant tumors in gynecology, it has been reported that the morbidity and mortality of cervical cancer ranks second among gynecological malignancies, second only to breast cancer [1, 2]. The number of new cases of cervical cancer has been increased every year around the world, and the onset age of cervical cancer has become much younger [3]. Cervical cancer is a malignant tumor, which occurs at the junction of squamous epithelial cells in the cervical vagina, or the transitional zone and columnar epithelial cells in the endometrium of the cervical canal [4]. The etiology is not totally clear, and it may be related to sexual behavior, frequency of deliveries and the infection of human papillomavirus [5, 6]. The early detection and treatment of cervical cancer is very essential to the prognosis of patients with cervical cancer.

In recent years, with the popularization of many effective screening methods, the overall mortality rate of cervical cancer patients has declined [7]. The clinical diagnosis mainly depends on clinical manifestations and gynecological examinations, which requires the combination of multiple auxiliary diagnostic methods including cervical cytology film, cervical multi-point biopsy. The International Federation of Obstetrics and Gynecology (FIGO) has developed a clinical staging standard for cervical cancer, which referred to the colposcopy pathological biopsy [8]. However, when comparing the early diagnosed stage with the patient's postoperative medical examination results, the stage of cervical cancer has been much underestimated [9–11]. More sophisticated radiological methods such as ultrasound, computer tomography and magnetic resonance imaging hasn’t been included in the stage of FIGO, but they have been the routine examination methods in clinical practice [12]. Many scholars [13, 14] have studied the characteristics of those examinations, but so far, there is no conclusion as to which is the best examination method for assessing cervical cancer staging. It’s necessary to conduct more studies to identify the role of those tools for early diagnosis of cervical cancer.

Previous studies [15–17] have reported that the transvaginal color Doppler ultrasound (TV-CDFI) can accurately reflect the size of the lesion, the extent of infiltration, and the blood supply, and it is widely used in the detection of cancers in breast and thyroid, but not in the cervical parts. Besides, it is well known that vascular patterns (such as density of vessels) is different for squamous and adenocarcinomas [18]. Therefore, it’s necessary to show the difference to provide insights into clinical treatment. Therefore, in order to make clinicians more clear about the blood supply of cervical cancer and facilitate communication between ultrasound doctors and clinicians, we attempted to conduct a preliminary investigation on the correlation between the Adler grade and clinical pathology of cervical cancer, to provide insights into the diagnosis and treatment of cervical cancer. The study design was: 1) participants: patients with cervical cancer; 2)intervention: TV-CDFI detection and Adler grading; 3)comparison: no applicable; 4)outcomes: Adler secores and antigens CA125 and CA199.

## Methods

### Ethical considerations

The study was approved by the Medical Research Ethics Committee of our hospital (No.20180038) and written informed consents were obtained from all the patients.

### Patients

Patients with cervical cancer diagnosed pathologically in our hospital from January 1, 2019 to December 31, 2019 were selected as study objects in this present study. The inclusion criteria were as follows: (1) there were surgical pathology or biopsy detections verifying small cell neuroendocrine tumors by our experienced clinicans, and the (FIGO) cervical cancer staging were used for severity differentiation [19]; (2) no history of other malignant tumors or history of radiotherapy and chemotherapy; (3) complete clinical data; (4) no history of cervical surgery such as we excluded patients undergone microwave, freezing, laser, electrocautery surgery. The exclusion criteria were as follows: (1) patients with congenital malformations of the uterus; (2) patients with coagulopathy; (3) patients with severe heart, liver, and kidney dysfunction; (4) patients with inability to cooperate with research or couldn’t follow-up.

### TV-CDFI detection

The color doppler ultrasound system (Philips E80) equipped with ultrasonographic probe DC5-8D were selected for ultrasound detection. Before the detection, all patients were asked to empty the rectum and bladder. When performing the doppler ultrasound detection, the patients took the lithotomy positions. Firstly we performed a transvaginal ultrasound examination of the uterus and bilateral accessory areas to determine the location, number, size, shape, boundary, echo of the cervical area and its relationship with surrounding tissues. Secondly, we chose the color Doppler blood flow imaging mode to observe the blood flow inside and around the cervical area, related parameters such the blood flow, cancer size have been calculated and collected. The staging and TV-CDFI done in the same setting and in different dates.

### Adler grade classification

Adler grade classifications were conducted based on the detected blood flow [20, 21]. We classified the blood flow signal of the lesion into four levels. Adler 0 refers to that no obvious blood flow signal; Adler I refers to 1 or 2 small blood vessels with a diameter of <1 mm are detected; Adler II refers to that 3 or 4 small blood vessels are detected; Adler III refers to that more than 4 blood vessels, or the blood vessels are intertwined into a network are detected. The Adler grade classifications were made by one radiologist and one gynecologist coherently, and if there were any disparity, further discussions were conducted for consensus.

### The detection of carbohydrate antigens CA125 and CA199

Before any drug treatment, 5 ml venous blood without anticoagulation in the early morning were collected from patients, the blood specimens were left at room temperature for 15min, then centrifuged it and separated the serum, then we stored it at environment with 5°C for further examination. The levels of serum CA125 and CA199 were detected by professional stall with electrochemical luminescence method. The electrochemical luminometer and detection kit used in the test were produced by Roche, and the operation process was strictly performed according to the instructions.

### Statistical analysis

SPSS 21 statistical software were used for data analysis. The measurement data for normal distribution was expressed as mean± standard deviation, and the measurement data for skewed distribution was expressed as median (P_25_, P_75_). The tumor size, CA125, CA199 were compared using the Kruskal-Wallis H rank test of multiple sample comparisons, of which Wilcoxon rank test was further used for pairwise comparisons. And t tests were conducted in the cancer size between pathological types or squamous cell carcinoma subtypes. Spearman correlation analysis was conducted for correlation analysis. And receiver operating characteristic (ROC) curves were drawn for evaluating the diagnosis value of Adler grade for cervical cancer [22]. p<0.05 was considered as being statistically different in this present study.

## Results

### The ultrasound characteristics of cervical cancer

**Fig 1:**
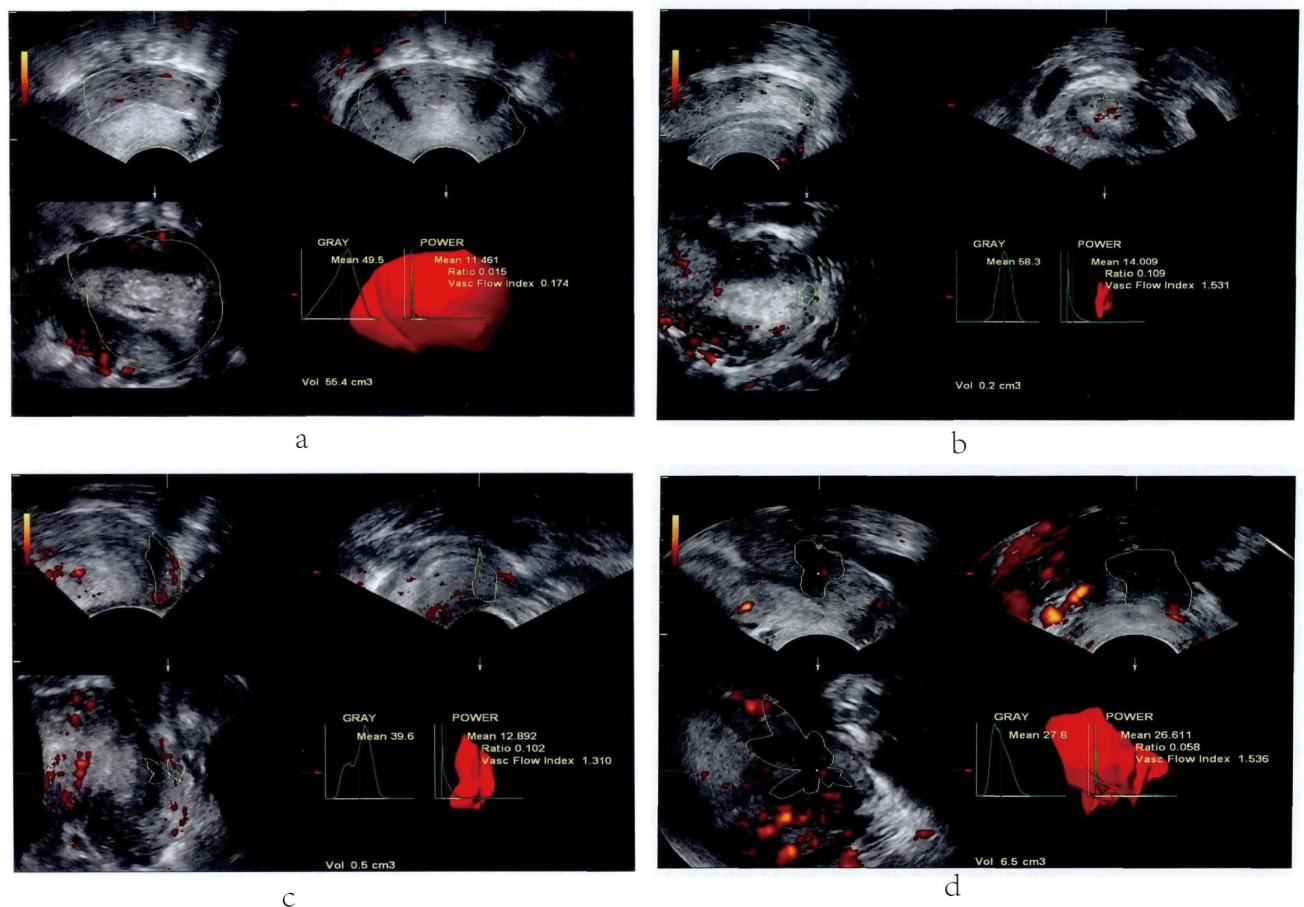
The Doppler ultrasound images on cervical cancer with different Adler grade. a, Adler 0; b, Adler I; c, Adler II; d, Adler III.

As Fig 1 presented, the ultrasound characteristics differed hugely among the ultrasound images with different Alder grades. For Alder 0, the cervical morphology was normal and no obvious blood flow signal were found in the images of color Doppler. For Alder I, the cervical morphology was slightly thickened, and more enhanced intra-cervical echo could be collected, one or two small spot-like blood flow signals could be detected. For Alder II, uneven or thickened cervical echo distribution could be detected, and strip blood flow signals at two to four locations could be seen. For Alder III, para-uterine and extrauterine invasion and blood metastasis could be found, and reticular blood flow signals could be detected.

### The distribution of Adler classification and clinical stage of cervical cancer

We identified 175 potential candidates firstly, and 13 patients were excluded. As Table 1 presented, a total of 162 patients were included for data analysis. With the increase of Adler severity classification, the clinical stage of cervical cancer increased accordingly.

**Table 1:** The distribution of Adler classification and clinical stage of cervical cancer.

Adler grade	FIGO grade			In total
	Grade I	Grade II	Grade III-IV	
Adler 0	0	0	0	0
Adler I	36	4	0	40
Adler II	31	48	0	79
Adler III	0	19	24	43
In total	68	70	24	162

### The comparison on the cancer size, CA125, CA199 with Adler grade and pathological parameters

As Table 2 showed, the cancer size differed significantly among the different Adler grades (p = 0.004), but there were no significantly differences in the level of CA125, CA199 among the different Adler grades (all p>0.05). There were significant differences in the level of CA125, CA199 between the squamous cell carcinoma and adenocarcinoma (all p<0.05), no significant difference was found in the cancer size between the squamous cell carcinoma and adenocarcinoma (p = 0.143). No significant differences were found in the cancer size, CA125 and CA199 between the keratinized and no-keratinized squamous cell carcinoma (all p>0.05). Furthermore, as Table 3 showed, the Adler grade was positively related with the FIGO staging, pathological type and squamous cell carcinoma subtypes of cervical cancer (all p<0.05), no correlations were found among the Adler grade and the cancer size, CA125, CA199 (all p>0.05).

**Table 2:** Relationship analysis on the cancer size, CA125, CA199 with Adler grade and pathological parameters.

Items	Cases	Cancer size(mm)	CA125[*M(P*_*25*_, *P*_*75*_*)* U/mL]	CA199[*M(P*_*25*_, *P*_*75*_*)* U/mL]
Adler grade				
Adler 0	0	0	0	0
Adler I	40	28.18±10.43	10.32(8.89, 17.13)	9.49(5.36, 14.39)
Adler II	79	36.04±12.59	14.53(8.83, 18.85)	10.83(5.89, 15.14)
Adler III	43	45.85±13.15	14.74(8.92, 19.06)	11.03(6.15, 15.99)
t/z		13.085	0.198	1.290
p		0.004	0.105	0.082
Pathological type				
Squamous cell carcinoma	141	35. 03±13. 35	13.05(8.19, 17.35)	9.32(5.25, 15.03)
Adenocarcinoma	21	35. 12±12. 22	18.16(9.36, 24.21)	11.56(5.89, 19.35)
t/z		10.317	1.589	0.864
p		0.143	0.007	0.013
squamous cell carcinoma subtypes				
Keratinized	43	35. 11±10. 52	12.76(8.05, 16.57)	9.31(5.33, 16.14)
Non-keratinized	98	35.02 ±11. 04	13.12(8.33, 17.01)	9.32(5.95, 16.09)
t/z		13.491	0.903	1.158
p		0.095	0.108	0.282

**Table 3:** Correlation analysis between Adler grade and clinicopathological parameters.

Clinicopathological parameters	r	p
FIGO grade	0.784	0.016
Cancer size	0.247	0.107
CA125	0.051	0.094
CA199	0.085	0.068
Pathological type	0.599	0.042
squamous cell carcinoma subtypes	0.603	0.017

### The diagnosis value of Adler grade for cervical cancer

**Fig 2:**
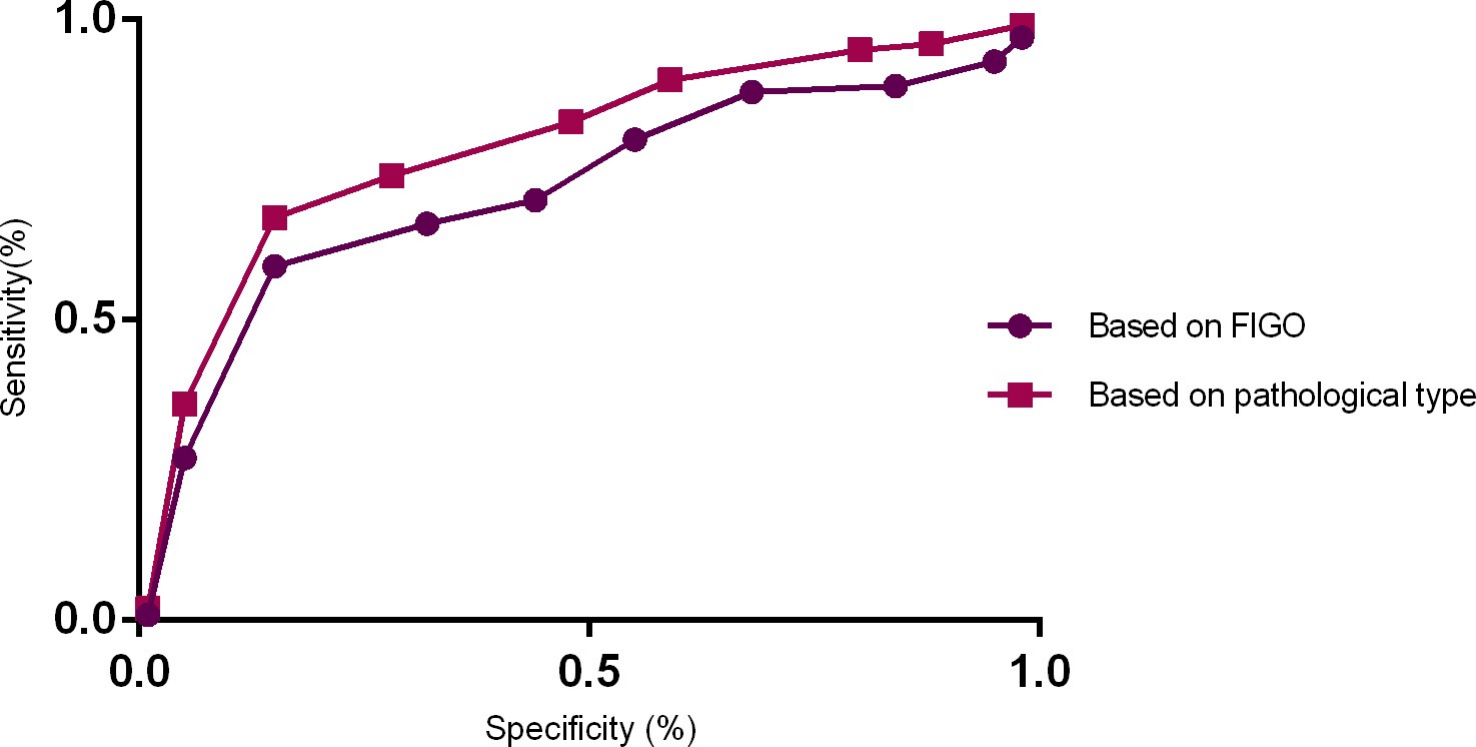
The ROC curve on the Adler grade for cervical cancer.

As Fig 2 showed, based on FIGO results, the area under the ROC curve of the cervical squamous cell carcinoma predicted by Adler grade was 0.811 (p<0.05), and its sensitivity, specificity, positive predictive value, negative predictive value, and Youden index were 54.67%, 89.53%, 74.14%, 85.63%, 0.48 respectively. And based on pathological results, the area under the ROC curve of the cervical squamous cell carcinoma subtype predicted by Adler grade was 0.762 (p<0.05), and its sensitivity, specificity, positive predictive value, negative predictive value, and Youden index were 52.46%, 88. 16%, 71. 04%, 79.36%, 0.42 respectively. Those results indicated that Adler grade could provide valuable reference for the diagnosis of cervical cancer.

## Discussions

TV-CDFI has the advantages of being non-invasive, cheap with convenient operation [16, 23]. It can not only clearly display the structure of each layer of the uterus, accurately locate and qualitatively diagnose the lesion, but also can observe the blood flow of detected area [24, 25]. Dynamic ultrasound is currently one of the most widely used and mature imaging methods in the diagnosis and treatment of obstetrics and gynecology diseases [26]. In this present study, the changes of cervical morphology and echo in Adler grade 0 to I were small, and Doppler blood flow signals were less displayed, but the blood flow signals in Adler grade II and III increased significantly, and the flake-like low echoes were seen inside. With the increase of Adler classification, cervical masses continue to increase, and color Doppler blood flow signals are also displayed. The results of this present study have indicated that Adler grade is expected to be useful to reflect the richness of blood flow in cervical cancer, which can provide important insights into the diagnosis and treatment of cervical cancer.

The occurrence, proliferation, invasion and metastasis of malignant tumors may largely depend on tumor neovascularization [27, 28]. Cervical cancer has characteristics of rapid proliferation and active cell division and growth, which is closely related to the proliferation of tumor vessels in it [29]. On color Doppler ultrasound images, there are often abundant different types of blood flow signals in tumor tissues, which correspond to its rich vascular network and are related to the special structure and blood flow characteristics of tumor blood vessels [30, 31]. The Alder grades are mainly concentrated on the blood flow signals, which to some extent can reflect the proliferation of vessels and the characteristics of microvessel density around the cervical cancer. Previous studies [32–34] have reported that the blood signals may predict the ability of tumor invasion and metastasis to a certain extent, which is consistent with the results of our study.

Squamous carcinoma is more common in the pathological and histological types of cervical cancer [35]. We have found that there was correlation between Adler grade and the histopathological type. The World Health Organization (WHO) classifies cervical squamous cell carcinoma into two subtypes, keratinized and non-keratinized, according to the presence of keratinized morphological features in the tissue [36]. Currently, the studies on the relationship of keratinized cervical squamous cell carcinoma and patient prognosis has been controversial. It’s been reported [37–39] that compared with non-keratinized cervical squamous cell carcinoma, the survival rate of keratinized cervical squamous cell carcinoma is significantly reduced, and it is not sensitive to radiotherapy, and the prognosis is very poor for untreated patients or advanced patients. Therefore, differentiate the various subtypes of squamous cell carcinoma of cervix is very helpful in clinical management.

In this study, Spearman rank correlation analysis showed that Adler grade was positively correlated with squamous cell carcinoma subtypes. Therefore, this technique is expected to be a reliable indicator for predicting the prognosis of patients with cervical cancer.

With the continuous progress of cancer marker research, the role of cancer related marker in the diagnosis and treatment of cancers has become increasingly important [40, 41]. Several studies have shown that cervical cancer is also closely related to the marker CA125 and CA199. It has been reported [42–44] that the carbohydrate antigens CA125 and CA199 are expressed at high levels in a variety of malignancies. In this study, the levels of CA125 and CA199 in patients with adenocarcinoma were significantly higher than those in patients with squamous cell carcinoma. The results were consistent with the findings of previous studies [45, 46]. Even through the Spearman correlation analysis shows that there is no correlation between Adler grade and CA125 and CA199, it may be explained that the samples in this present study is not large enough to powerfully detect the difference, future studies with larger population samples are warranted to identify the role of CA125 and CA199 in cervical cancer.

Several limitations must be concerned in this present study. Firstly, the sample size of this study is not large enough, and the clinical staging is relatively rough, biases may be existed in this present study. Secondly, we did not use the cervical cancer specific markers such as squamous cell carcinoma tumor marker for reference, it should be further elucidated in the future studies. Thirdly, it must bring to our attentions that the subjective factors in the Adler classification, different operators and different parameter settings may bias the test results. However, we have performed related trainings on the TV-CDFI before this study to avoid unnecessary errors in the process of ultrasound detection and analysis.

In conclusion, the results of this study have favored the value of Adler grade in the diagnosis and treatment of cervical cancer. With the continuous development of ultrasound technology and popularization of clinical ultrasound applications, Adler grade should be promoted in the application of color Doppler ultrasound for the diagnosis and treatment of cervical cancer.

## Supporting information

S1 Checklist (DOCX)

S2 Checklist (DOCX)

## List abbreviations

FIGO: International Federation of Obstetrics and Gynecology
TV-CDFI: transvaginal color Doppler ultrasound
WHO: World Health Organization
